# Influence of Biodegradable Polymer Properties on Antifouling Paints Activity

**DOI:** 10.3390/polym9020036

**Published:** 2017-01-25

**Authors:** Marion Loriot, Isabelle Linossier, Karine Vallée-Réhel, Fabienne Faÿ

**Affiliations:** Laboratoire Biotechnologie et Chimie Marines, Université Bretagne Sud, EA3884, LBCM, IUEM, 56321 Lorient CEDEX, France; marion.loriot@univ-ubs.fr (M.L.); isabelle.linossier@univ-ubs.fr (I.L.); karine.rehel@univ-ubs.fr (K.V.-R.)

**Keywords:** antifouling paint, hydration, degradation, erosion, bacteria, diatoms, biofilms

## Abstract

The development of new antifouling paints requires understanding the parameters involved in antifouling activity and to develop new analytical tools for their evaluation. A series of biodegradable poly(ε-caprolactone-*co*-δ-valerolactone) copolymers varying by molecular weight and composition were synthesized, characterized and formulated as antifouling paints. The physico-chemical properties such as hydration, degradation, erosion and lixiviation of paints were studied. Microfouling (bacteria and microalgae) was observed by microscopic observations in a short delay, whereas macrofouling colonization was observed by visual inspection during one year. The antifouling activity of paints was modified by varying the composition and molecular weight of copolymer. The crystallinity appears to play a major role in antifouling activity, however the involvement of other properties such as hydration, degradation or erosion remains difficult to understand. Confocal laser scanning and scanning electron microscopes were used for the evaluation of antifouling paints. Results show that microalgae seem to be a pertinent indicator of antifouling activity.

## 1. Introduction

Biofouling is a multi-stage process, which includes adsorption of dissolved organic molecules, colonization by prokaryotes, eukaryotes and recruitment of invertebrate larvae and algae spores [1]. The presence of microfouling such as diatoms and bacteria, their composition but also the substratum properties are among the most important factors that determine the attachment of much larger fouling organisms [2]. Prevention of fouling includes continuous maintenance involving intensive labor and use of antifouling strategies. Most current protective systems are based on biocide-containing antifouling paints [3,4]. These have traditionally relied upon the release of biocides (organic biocides called booster biocides and copper) to kill or inhibit the growth of fouling organisms [5]. In recent years, other antifouling polymer coatings, without any release mechanism or with biocides having short environmental half-lives are being developed [6,7,8,9,10,11,12].

Our research group focuses on the use of biodegradable polymers derived from polycaprolactone. In previous studies, a biodegradable binder, the poly(ε-caprolactone-*co*-δ-valerolactone) (P(CL-VL)), has been described [13,14]. This copolymer, with 80% and 20% of caproic and valeric units, respectively, and a molecular weight of 20,000 g/mol average, has shown promising results as antifouling binder. The polymer possesses the different essential characteristics: solubility in aromatic solvents, compatibility with fillers and several active molecules and biocides, controlled degradation and molecule release [14,15]. The corresponding paint has shown a homogeneous erosion similar to paints based on polyacrylic and rosin binder [16]. The macrofouling was completely absent from paint after one year of immersion [13].

The development of new antifouling systems requires the development of dedicated evaluation protocols. Field tests are more frequently used to evaluate antifouling coatings because they are accurate and credible. However, it is also a matter of fact that field tests take quite a long immersion time. Hence, it is necessary to develop reliable and rapid assay methods to identify promising coatings [17]. Two ways are currently followed. The first one concerns the evaluation of many properties suspected to be involved in antifouling activity [16,18,19]. Four parameters are described: hydration, degradation, erosion and biocide release. Nevertheless, several studies have shown the complexity of these mechanisms and the difficulty of relating them to each other and to antifouling activity [16,17,18]. The second one consists in studying the efficiency of coatings, using in vitro conditions, against selected organisms such as bacteria, diatoms, spore (*Ulva* sp.) and barnacles (*Balanus amphitrite*) [17,20,21]. Despite many advantages (speed, reproductibility, and independence from the season or the disposal site), bioassays can never fully mimic environmental conditions.

Furthermore, recently, several studies have focused on biofilm communities present on fouling release coatings and to a lesser extent on antifouling paints immersed in natural seawater [22,23,24,25,26,27,28]. Commercially biocidal coatings exhibit high efficiency in preventing macrofouling and significantly resist, but not totally inhibit, the formation of microfouling [29,30,31]. It has been shown that coatings subjected to identical immersion conditions reflected differences in microbial fouling communities that were attributed to the coating type, its functions and its biocide composition [22,26,29]. The quantitative and qualitative description of biofilms (i.e., number of species present, their thickness and biomass) is important for better predictive studies allowing the design of new antifouling systems [25]. Indeed, the growth of biofilms on biocidal coatings: (i) interferes with the performance of these coatings by altering the release rates of biocides leaching resulting in the loss of properties; and (ii) increases shear stress and drag, leading to increased fuel consumption [19].

In this context, the present study proposes to evaluate five P(CL-VL) binders varying by their molecular weight and composition. The resulting coatings have been characterized in terms of antifouling properties: (i) parameters involved in the activity; and (ii) performance of paints against the biofouling. Furthermore, this work has studied the interest to evaluate the coatings against a wide range of marine organisms (micro- and macrofouling) to assess the in situ antifouling coating performances in real conditions.

## 2. Materials and Methods

### 2.1. Copolymerization

Copolymers (20 g average) were synthesized by bulk ring-opening polymerization (ROP) of ε-caprolactone (CL) (Aldrich, Saint Quentin Fallavier, France) and δ-valerolactone (VL) (Acros, Geel, Belgium) in different proportions under nitrogen atmosphere. Octanol (Aldrich) and tin(II)octoate (98%, Aldrich) was used to promote copolymerization with an initial molar [octanol]/[SnOct_2_] ratio of 2. After degassing at 140 °C (10 min), the reaction was carried out 6 h at 140 °C, and stopped by quenching in an ice bath. A mechanical stirrer was performed to homogenize the mixture. The polymers were recovered by the dissolution/precipitation method with THF (Fisher, Illkirch, France) as solvent and petroleum ether (Fisher) as no solvent. Polymers were recovered by filtration and drying at room temperature.

### 2.2. Paints Preparation

Paints were formulated following the composition detailed in the Table 1. All the ingredients were dispersed under vigorous agitation (1500 rpm) for 20 min, and then filtered. A layer of coating was deposited with an automatic film applicator (ASTM D823 Sheen instrument, Saint Denis, France) on a polycarbonate support. The specimens were dried at 20 °C until they achieved a constant weight (dried thickness: 80 μm average).

### 2.3. Hydration

Pieces of paints (1 cm^2^) were cut off in order to quantify water amount present in the film. They were immersed into demineralized water at 20 °C in a thermostatically controlled bath. The films were analyzed by Karl Fisher titration (Methrom, Villebon Courtaboeuf, France) as described previously [16].

### 2.4. Degradation

Three replicates of each specimen were withdrawn from the degradation medium (demineralized water at 20 °C), dried, and were monitored by Gel Permeation Chromatography (Agilent Technology, Santa Clara, CA, USA). Degradation rate was defined as followed: Degradation = (*M*_n(t)_/*M*_n(0)_) × 100 where *t* and 0 represent the molecular weight at *t* and initial time, respectively.

### 2.5. Erosion

Erosion was estimated with a direct method called the “touching” method. It consisted in rubbing the surface of paint. The resulting prints on fingers enabled the examiner to estimate the erosion rate by comparison between paints [16].

### 2.6. Lixiviation Tests

Lixiviation tests were conducted according to a normalized protocol (AFNOR, NF ISO15181). Immersion and test procedures were carried out as previously described [32]. Synthetic seawater was composed as following: NaCl (24.5 g/L, Fisher), MgCl_2_·6H_2_0 (5.20 g/L, Acros), Na_2_SO_4_ (4.09 g/L, Aldrich), CaCl_2_ (1.16 g/L, Carlo Erba), KCl (0.695 g/L, Fisher), NaHCO_3_ (0.201 g/L, Carlo Erba), and H_3_BO_3_ (0.027 g/L, Carlo Erba, Val de Reuil, France). Surrounding water was analysed at each sampling dates and changed in order to avoid the risk of medium saturation. The releases of zinc pyrithione and copper thiocyanate were quantified by atomic absorption spectroscopy.

### 2.7. Field Immersion Test

#### 2.7.1. Characteristics of Immersion Site

After drying, duplicate panels were exposed in natural seawater, at a depth of 50 cm (Atlantic Ocean, W 37°43′08,5″ N 3°22′W, Larmor Plages, France). The study began in March 2014. Inspections were done weekly. A commercial paint (A4Tspeed) was supplied by Nautix Compagny (Guidel, France). It was used as performing references. Paint without biocides was used as negative control. It was formulated as described in the Table 1.

#### 2.7.2. Microfouling Observations

The analytical procedure was used to obtain information about the microscopic colonization of paints during immersion as described previously [33]. Paint formulations were applied onto polycarbonate panels (4 cm × 2 cm) with a dry thickness of about 80 μm. Coupons were analyzed after 6 weeks and 3 months and observed by SEM and CLSM microscopies. The data of antimicrofouling performance were ranked from the best to the poorest, and the significance test was conducted using one-way analysis of variance (ANOVA).

#### 2.7.3. Macrofouling Observations

Paints were applied onto panels (10 cm × 10 cm). The Antifouling (AF) performance was assessed according to a modified protocol of the French Standard (NFT34-552 September 1996) [34]. The percentage of surface covered by fouling and the type of fouling organisms are recorded (Table 2). Antifouling coatings were classified using an efficiency parameter N which is expressed as *N* = Σ*I*·*G* where *I* stands for the intensity of fouling which is defined by the percentage of fouling on the surface and *G* the severity of fouling.

### 2.8. Characterization Technique

#### 2.8.1. Molecular Weight Measurements

Average molecular weights were determined by Gel Permeation Chromatography (GPC) using a Merck pump L-7110 with two columns PLgel (mixte C, 3 μm and mixte D, 5 μm) in series from Polymer laboratories, and a Sedex DEDL detector (55 °C, 2 mbar). THF was used as eluent at a flow rate of 1.0 mL/min and the injection volume was 20 μL. A calibration curve was generated with polystyrene standards (Easical PS-2) purchased from Agilent Technologies (Santa Clara, CA, USA). Samples concentration was 1 mg/mL. Data were analyzed by Cirrus software (Agilent Technology, Santa Clara, CA, USA).

#### 2.8.2. Thermal Analysis

Thermal analysis was conducted by differential scanning calorimetry (DCS) using a Mettler-Toledo DSC 822 (Mettler Toledo, Viroflay, France). The samples were scanned from 25 to 80 °C with a heating rate of 20 °C/min then cooling at −100 °C at 3 °C/min, and scanned again from −100 to 80 °C with a heating rate of 3 °C/min. The glass transition temperature (*T*_g_) was taken as the midpoint of the transition. Melting point (*T*_m_) was taken as the summit of melting peak and ∆*H* was calculated from the area of the endothermic peak after the second run.

#### 2.8.3. Nuclear Magnetic Resonance Spectroscopy

The 500 MHz ^1^H NMR spectra of the samples were recorded at room temperature in CDCl_3_ on a 500 Bruker spectrometer (Bruker Biospin SAS, Wissembourg, France).

#### 2.8.4. Karl Fisher Titration

A coulometer Metrohm KF 831 (Methrom, Villebon Courtaboeuf, France) equipped with an Metrohm 860KF Thermoprep (*T* = 150 °C) was used under an air flow at 80 mL/min. The reactant was Hydranal-Coulomat AG.

#### 2.8.5. Atomic Absorption Spectroscopy

Samplings (30 mL) were acidified with a solution of nitric acid (1%) to dissolve all degradation products. The spectrometer was a SAA Analyst 200 Perkin Elmer (Perkin Elmer, Villebon sur Yvette, France). Wavelength used was 325 and 314 nm for copper and zinc respectively. The standard curve was realized from 0.2 to 1 mg/L in nitric acid 1%. The zinc and copper standard solution (1 g/L) were furnished by Varian.

#### 2.8.6. Scanning Electronic Microscopy

Coupons were immersed in 3% glutaraldehyde solution overnight at 4 °C and then dehydrated by several washings: phosphate buffer (pH = 7.35, 10 min, 3 times); ethanol 70% (10 min, 3 times); ethanol 90% (10 min, 3 times) and absolute ethanol (10 min, 3 times). Then, the samples were desiccated by the carbon dioxide critical point method. Samples were made conductive with a gold deposit. Samples have been observed on a JEOL 6460LV microscope (JEOL, Croissy sur Seine, France). Images were taken with a magnification of 1000, under an acceleration voltage of 7 keV, from secondary electrons.

#### 2.8.7. Confocal Laser Scanning Microscopy

Adherent cells on paints were stained by adding Syto 9 stain (5 μM, Molecular Probes), which is a fluorescence-based assay for cell viability (excitation and emission λ, 485 and 498–550 nm, respectively). They were observed after a 10 min-incubation period. Micro-algae were observed in red by autofluorescence (excitation and emission λ, 633 and 650–670 nm, respectively). Images were captured with CLSM using a TCQ-SP2 (Leica Microsystems, Nanterre, France). The thickness of the biofilm (μm) and the biovolume (μm^3^/μm^2^) were measured using COMSTAT software (www.comstat.dk) [35]. Results were representative of 3 observation sites. The points were randomly chosen.

## 3. Results and Discussion

### 3.1. Characterization of Biodegradable Binders

**Scheme 1:**
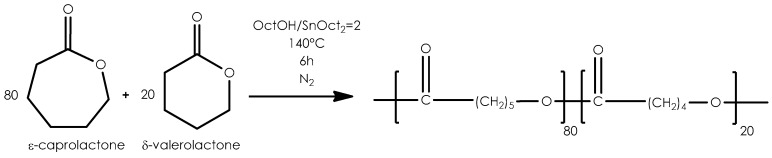
Copolymerization of α-caprolactone (CL) and δ-valerolactone (VL).

A series of copolymers were prepared varying by their molecular weight and composition. P(CL-VL) polymers were synthesized in bulk by ring opening polymerization of ε-caprolactone and δ-valerolactone as promoted by octanol and Sn(Oct)_2_ upon a coordination-insertion mechanism (Scheme 1, Table 3) [36]. The resulting copolyesters were recovered at high yields excepted for polymer 69/31L. The molecular weight (*M*_n_) of the copolymers synthesized ranged around 12,000 g·mol^−1^ (polymers 82/18L, 69/31L, and 22/78L), 25,000 g·mol^−1^ (polymer 82/18M) and 40,000 g·mol^−1^ (polymer 82/18H) with a polydispersity between 1.5 and 1.7.

The crystallinity of copolyester was investigated using DSC by determining melting temperature (*T*_m_) and melting enthalpy values (∆*H*_m_). Results are shown in Table 3. The melting temperature value increased with the molecular mass, as is already known [37,38]. Nevertheless it remained constant (30 °C average) regardless the composition (polymers 82/18L, 69/31L, 22/78L). Previous studies have proved a clear decrease in the melting temperature in function of the composition [39,40,41]. Differences could be explained by the molecular weight of the copolyesters (12,000 g/mol average) lower than published data.

P(CL-VL) polymers were highly crystalline over a broad composition range [41]. Nevertheless, the melting enthalpy values varied with the valerolactone composition. As previously shown [13,40,41], ∆*H*_m_ dropped from 60 J·g^−1^ for polymer 82/18L to 36 J·g^−1^ for polymer 69/31L. The presence of VL units perturbed the organization of macromolecular chains [13]. In the same way, ∆*H*_m_ increased with molecular weight: the values varied from 60 to 81 J·g^−1^ as molecular weight increased. Hence, Table 3 shows clearly that the crystallinity of P(CL-VL) copolyesters was reduced with co-monomer ratio (69/31L < 22/78L < 82/18L) and increased with molecular weight (82/18L < 82/18M < 82/18H).

### 3.2. Properties of Biodegradable Paints

The major properties of the paints come from the binder [42,43]. Some potential parameters involved in antifouling activity of erodible paints are the hydration, the erosion and the biocide release [10,12,16,32,44]. These properties are suspected of being related to the binder crystallinity [12,39,41,45]. To perform these aspects, the previous copolymers were formulated as paints (Table 1). The commercial biocides are the same molecules for every formulation. In this condition, only the binder could influence the antifouling properties [16,18,19].

#### 3.2.1. Hydration

It is known that water uptake is dependent of a number of properties of the binder [9]. To quantify water absorption, a Karl-Fisher coulometer (Methrom, Villebon Courtaboeuf, France) was used (Table 4). Only one paint was distinguished by a different kinetics (69/31L). This one reached a saturated hydration rate after 14 days of immersion (20.6%), then the hydration remained constant. Contrariwise, the other coatings exhibited a real hydrophobicity with a progressive hydration. Water uptake remained less than 10% after 14 days of immersion. After this time, the hydration continued to rise during eight months. Nevertheless, the rate reached was not the same for all paints.

Paints based on copolymers containing the same composition but varying in molar masses (82/18L, 82/18M, and 82/18H) showed similar hydration rates ranked between 12.9% and 16.4%. Concerning, the influence of composition, differences were more pronounced: the hydration rates varied from 9.7% to 16.4%.

A relation between the hydration of paints and the crystallinity of polymers was difficult to establish. This result can be explained by the presence of additives and fillers that modify the behavior of polymers during immersion [12,13] and more precisely disturb the macromolecular chains organization. However, some results showed that the hydration of paints can be increased with a significant decrease of crystallinity of the binder: paint 69/31L had a hydration rate of 20.6% and the corresponding polymer a melting enthalpy of 36 J·g^−1^, whereas paint and copolymer 82/18H had 4.6% and 81 J·g^−1^ for hydration and melting enthalpy, respectively. This difference in hydration could be explained by the morphology of the binder. The more the binder is crystalline the more the crystalline zones are numerous compared to amorphous zones. Upon hydration of the coating, it is more difficult for water molecules to penetrate the crystalline areas.

#### 3.2.2. Degradation

During their immersion, paints are indeed submitted to profound changes (degradation principally) [12]. These transformations could modify strongly the film properties and influence paint erosion and biocide release. Moreover, the rate of hydrolytic degradation of ester linkages in polymers is affected by a multitude of factors such as pH and temperature, water absorption and crystallinity [46]. The degradation of polymers formulated as paints was evaluated by gel permeation chromatography as shown in Table 4. Coatings based on copolymers containing 18% valerolactone that were the more crystalline showed insignificant degradation whatever the molar mass. This data correlated those obtained in previous studies [12,39,41]. The presence of crystalline domains slowed down dramatically the hydrolytic degradation of the polymers [47]. In the crystalline domains, the polymer chains were more packed, water found more difficult to penetrate. Contrariwise, the paints based on copolymers whose composition varies showed different behaviour. The degradation increased with VL content: paint 22/78L reached 26% degradation. The decrease of crystallinity (∆*H*_m_ decrease from 60 to 36 J·g^−1^) by incorporating up to 31% of VL accelerated the copolymer degradation. These results were in accordance with the degradation studies of Lin [39] and Fernandez et al. [41]. Moreover, the macromolecular chains degradability appeared to be affected by the valerolactone content. The data may be explained by the higher degradability of valeric acid unit sequences and the higher solubility in water of valeric acid (497 mg/L) comparatively to caproic acid (108 mg/L). The CL unit has an identical structure to VL but has one straighter methylene groups sandwiched between the ester groups of the polymer chain. Hence, the fraction of hydrolytically ester bonds was richer in the case of the polymer 22/78L than the polymer 82/18L.

#### 3.2.3. Erosion

The erosion can promote a cleaning of the surface by a mechanical action with the release of fillers. High erosion rate values lead to excellent antifouling properties but with short duration. Contrariwise, low erosion rates limit the release of biocides and their concentration is not sufficient to prevent the development of fouling. Thus, the study of erosion of erodible paints is necessary to understand antifouling activity of marine paints. The test was realized on natural site by relative comparison between paints.

**Figure 1:**
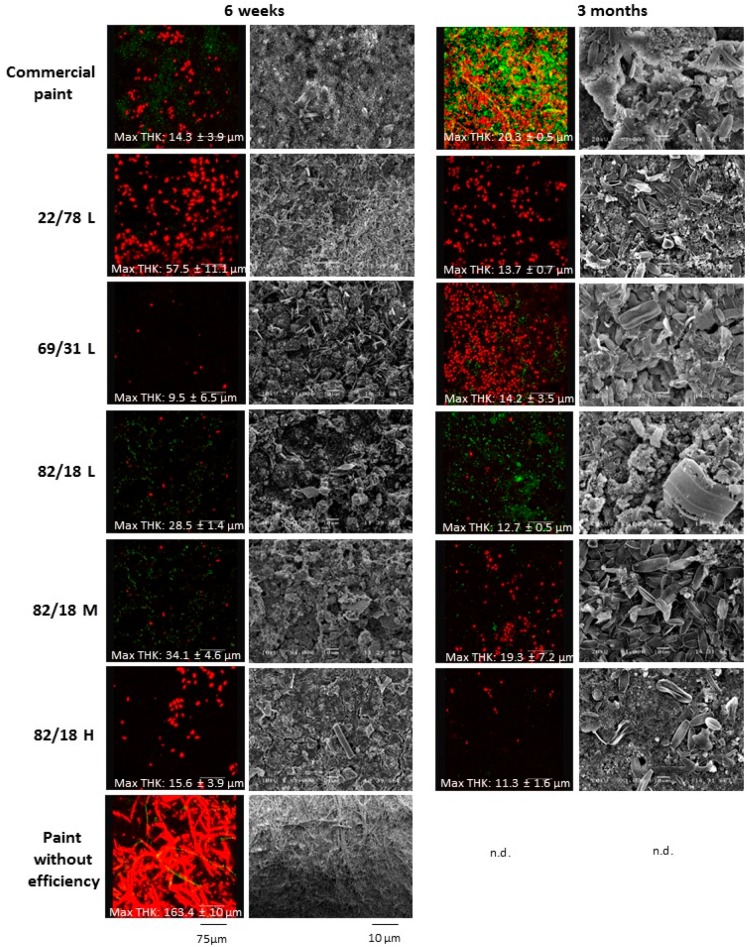
Microfouling development onto paints immersed in Ocean Atlanti.

The erosion rate of P(CL-VL) paint was previously investigated [16]. Experiments showed that the erosion was in bulk with rate of 0.45 μm/week (molar composition: 83/17, *M*_n_ = 21,700) lower than commercial ablative paint (2.50 μm/week). However, present experiments showed that the erosion was modulated by varying the molar ratio of comonomer and the molecular weight. After eight months, paint 69/31L was the most erodible, followed by paint 82/18L. A rank can be proposed from the least erodible to most erodible: 82/18H < 22/78L~82/18M < 82/18L < 69/31L. The observation of SEM micrographs showed the surface morphologies after six weeks of immersion (Figure 1). For 82/18H paint, the surface appeared smooth, dense, without large porosity. Inversely, the 82/18L presented a granular surface with less cohesion. Erosion seemed to be correlated with hydration and crystallinity.

#### 3.2.4. Biocide Release

**Figure 2:**
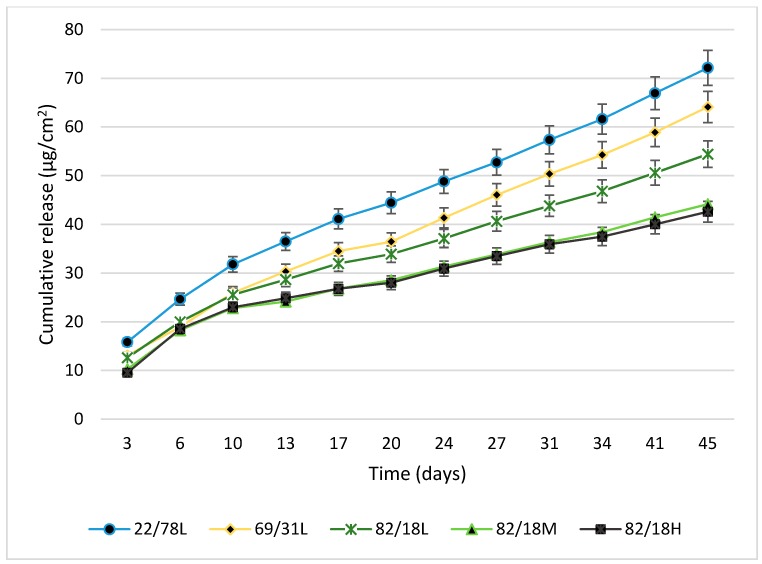
Release of zinc pyrithione from P(CL-VL) paints.

Zinc pyrithione (ZPT) and copper thiocyanate were chosen for this study. The first one has been presented as an environmentally-friendly alternative to tributyltin, being photodegradable within a short time frame [9,48]. The molecules release was quantified following normalized protocols. The total amount of zinc pyrithione and copper thiocyanate from paints during immersion for 45 days in artificial seawater was determined. Cumulative release of zinc pyrithione and copper thiocyanate during immersion was determined by atomic absorption spectroscopy. Copper thiocyanate was not detected in the surrounding water: the rate of copper leaching was lower than the detection limit. Contrariwise, zinc pyrithione was quantified (Figure 2). These observations can be explained by: (i) their difference of water solubility, i.e., zinc pyrithione is more soluble in water (8 mg/L, 20 °C) than copper thiocyanate (8.43 × 10^−3^ mg/L, 20 °C); and (ii) their affinity states in paints [49].

Concerning zinc pyrithione, macromolecular structure (composition and molecular weight) appeared to influence the biocide release. Paints based on copolymers 82/18M and 82/18H, possessing the same behavior in term of hydration and degradation, showed the same leaching profile. Nevertheless, the biocide release seemed to be partially related to molecular weight: an increase of release was observed with the decrease of molecular weight. For the paint based on copolymer 82/18L, the release of zinc pyrithione was higher than for the two other coatings: 55 μg/cm^2^ after 45 days of immersion The comparison between polymers with variable compositions seemed to show a link between biocide release and degradation: paint 22/78L was more susceptible to degradation and its release of biocide was higher. For the same molecular weight (10,000 g·mol^−1^ average), the biocide release was linked with the increase of VL units, sensitive to hydrolysis.

The average release rate until 10 days was found to be between 6 and 8 μg/cm^2^/day, whereas these rates decreased between 10 and 45 days (2–4 μg/cm^2^/day). These values were conventional for an erodible paint [50].

### 3.3. Influence of Binders on Antifouling Properties

The several binders were subjected to the evaluation of their antifouling activity. The influence of composition and molecular weight on antifouling paints activity has been examined and compared to a commercial reference paint and a paint devoid of antifouling activity (negative paint). Coatings were immersed in Atlantic Ocean and observed for their antimicro- and antimacrofouling properties.

#### 3.3.1. Paints Activity against Micro-Fouling

**Figure 3:**
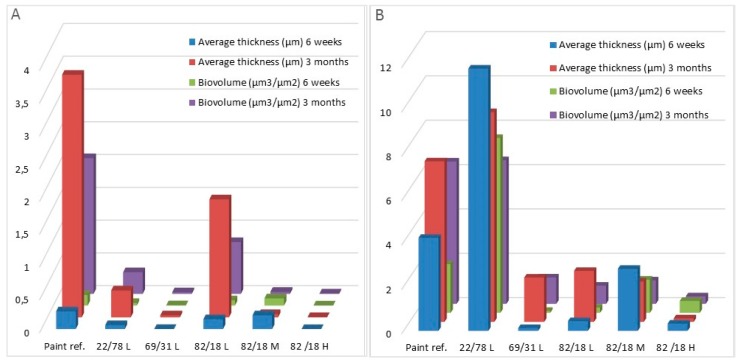
Quantification of: (**A**) bacteria; and (**B**) microalgae in biofilms on paints after six weeks and three months of immersion.

Coatings were immersed during three months and observed by CLSM and SEM at two times (six weeks and three months) (Figure 1). The biofilm was quantified by COMSTAT analysis to obtain biomass and average thickness of bacteria (Figure 3A) and microalgae (Figure 3B) on paints (Table 5). Bacteria and microalgae were observed on all paints. Nevertheless, some differences were observed. Negative paint showed a denser and thicker biofilm than other paints.

The bacterial biofilm that developed on all P(CL-VL) paints after three months was noticeably reduced versus reference paint: biomass and average thickness values were always lower than for the reference paint.

For microalgae biofilms, the same result was observed except for those of 22/78L: data of biomass (7.89 μm^3^/μm^2^) and average thickness (11.83 μm) quantified on this paint after six weeks were higher than for reference paint (2.2 μm^3^/μm^2^ for biomass and 4.2 μm for average thickness). After three months, both paints showed the same biomass and average thickness values.

Moreover, differences were observed depending P(CL-VL) characteristics. The modulation of molecular weight and composition influenced the anti-microfouling properties but different activities were observed against bacteria or microalgae. After three months, in the case of bacteria, P(CL-VL) paints can be ranked in the order: 82/18H = 69/31L = 82/18M > 22/78L > 82/18L (*p* = 0.196, 0.589, 0.002, and 0.006, respectively, for bacteria biomass, and *p* = 0.156, 0.423, 0.003, and 0.049, respectively, for bacteria average thickness), whereas, for microalgae, a different behavior was observed. The paint based on polymer 22/78L showed a lower activity (*p* = 0.0001 for microalgae biomass and average thickness) than coatings based on 69/31L = 82/18M = 82/18L = 82/18H (*p* > 0.05 in all cases). For example, the paint based on polymer 82/18L showed a good activity against microalgae but its antibacterial activity was greater. The paint based on the polymer 22/78L showed an inverse trend. The paint based on polymer 82/18H seemed to present the best activity against bacteria and microalgae closely followed by 82/18M and 69/31L.

The lower efficiency of 22/78L against microalgae biofilm can be explained by the high zinc pyrithione release (Figure 1) rapidly decreasing the efficiency of this paint [51]. Another hypothesis concerns the nature of surface [13]. This copolymer contains a higher content of VL: the microalgae can adhere on this surface preferentially due to a greater affinity [52].

#### 3.3.2. Paints Activity against Macro-Fouling

**Figure 4:**
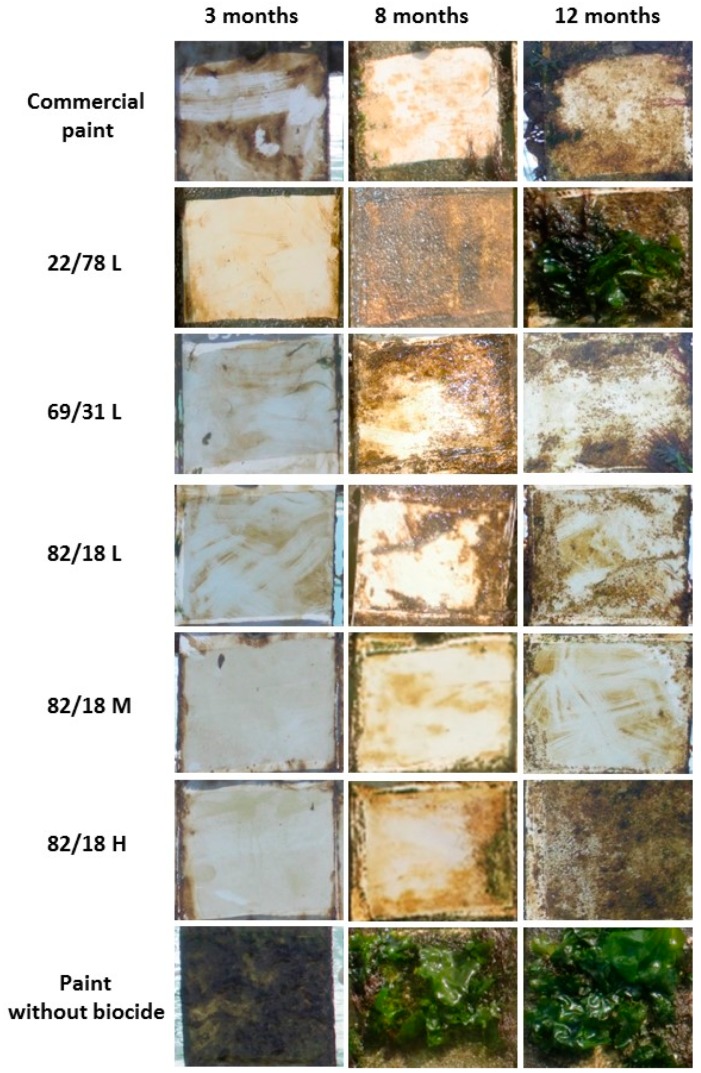
Visual inspection of paints after three and eight months of immersion.

**Figure 5:**
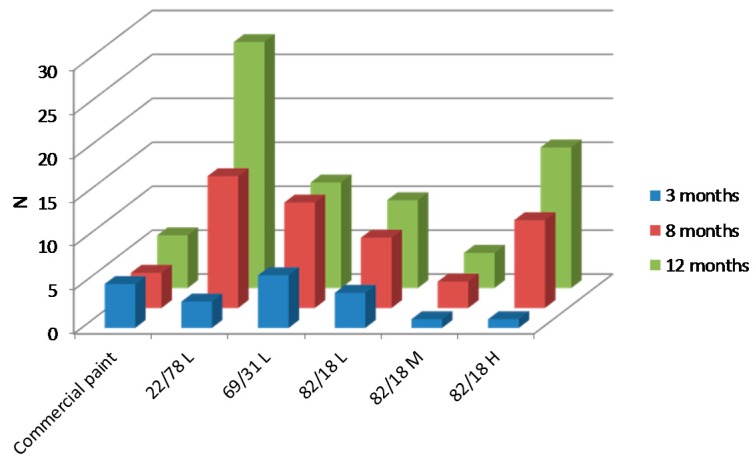
Values of the AF efficiency *N* for P(CL-VL) based paints immersed for 3, 8 and 12 months in Atlantic Ocean. Commercial paint was used as positive controls.

Paints were immersed during 12 months for visual inspection (Figure 4) and an efficiency factor (*N*) was determined at each observation time (Figure 5). All paints showed lower *N* values than negative paint (*N* = 15, 30 and 35 after 3, 8 and 12 months of immersion, respectively) confirming its deficient activity. Up to 12 months of immersion, no adherent macrofoulers were observed on P(CL-VL) paints (except for paint 22/78L, which exhibited green algae). Coatings showed slime on the surface, which could be removed by wiping. The *N* values assessed from inspection at three months of immersion were lower than reference paint (except for paint 69/31L which was comparable). Results corroborated with paints activity against microfouling (Figure 3).

The determination of efficiency from *N* values at three months showed a better efficiency for reference paint than other coatings (Figure 5), while the average biofilm thickness was higher. The study of *N* values after three months cannot be connected to the colonization of microfouling at the same date.

After 8 and 12 months, the efficiency was lower for P(CL-VL) paints: N values were higher than for the reference paint. However, one paint (82/18M) maintained N values (*N* = 1, 3 and 4) lower than reference paint (*N* = 5, 4 and 6) after 3, 8 and 12 months, respectively. After 12 months, a rank for the N value can be proposed: 82/18M < 82/18L < 69/31L < 82/18H < 22/78L. Thus, paint 22/78L confirmed its low efficiency (*N* > 25). This coating was already differentiated after three months by showing an important amount of microalgae compared to other coatings. Hence, P(CL-VL) with appropriate physico-chemical properties can be considered an appropriate polymer for binder in antifouling paints. Particularly, P(CL-VL) paints can be active against green algae and barnacles.

#### 3.3.3. Influence of Polymer Characteristics on Colonization

The object of this work was to investigate the direct relationship between different characteristics (molar mass, and composition) of the binders (polymers) and the antifouling activity of the corresponding paints by also investigating certain proprieties of the coatings (hydration, degradation, erosion, and biocide release). The results clearly established that the properties of the binder affect the performance of the paint and that both erosion and biocide release are discriminating properties. It was possible to classify the different polymers according to the effectiveness of their corresponding paint. The best binder being copolymer 82/18M and the worst being copolymer 22/78L. The classification could be explained as follows: the binder 22/78L led to a paint with an adequate erosion but a too large biocide release limiting its duration. This leaching seemed to be the consequence of a degradation of the sensitive VL units. The copolymer 69/31L gave a coating with excessive erosion, adversely affecting the film durability. Copolymer 82/18L led to similar results. Only polymer 82/18 M combined adequate erosion and release, copolymer 82/18H not being erodible.

The second conclusion that could be drawn from the results obtained was that it is difficult to link the properties of the polymers like crystallinity to those of the paints (hydration, degradation, erosion and biocide release) and to link the properties of the paints with one another. The impact of the formulation, of the interactions between charges, of the interactions between binder and fillers is probably important in the phenomena observed.

Although the relationships between the characteristics of the polymers and the properties of the corresponding paints are complex, it is useful to identify factors to be controlled for the development of new erodible binders. Given the results obtained, it seems that the crystallinity and the presence of easily hydrolysable groups are two major factors.

#### 3.3.4. Link between Micro- and Macrofouling

Results about activity of P(CL-VL) paints showed different behaviors against micro- or macroorganisms. This result has already been observed in several studies [6,14,17]. The observation and quantification of microfouling developed on paints after a few weeks of immersion was not sufficient to know without ambiguity the efficiency of antifouling paint. Nevertheless, these observations can give first information on paint efficiency.

The worst paints can be distinguished. For example, 22/78L and negative paints showed the most microalgae biofilm (microalgae biomass up to 6 μm^3^/μm^2^) and lower activity against macrofouling (*N* > 25).

The best paints can be distinguished. Paints based on 69/31L, 82/18L, 82/18M and 82/18H showed lower biofilm (microalgae biomass between 0.34 and 1.19 μm^3^/μm^2^) and higher activity against macrofouling (*N* < 15).

Microalgae seem to be more sensitive and thus a better indicator of antifouling activity than bacteria. The four paints that were active against microalgae were efficient against macrofouling. Results corroborated Zhang et al. [17] in the case of FR coatings. However, the complexity of the relationships between fouling and biofilm needs caution to avoid over-generalization [53].

## 4. Conclusions

The research focused on the development of optimized P(CL-VL) copolymers as binders for antifouling paint. Results demonstrated the influence of binder properties (molecular weight and composition) on properties involved in antifouling activity (hydration, erosion, degradation and biocide release). Nevertheless, they confirmed the difficulty of linking these different parameters. The observations were not always sufficient to understand and connect all the results. Only the copolymer crystallinity and the presence of hydrolysis sensitive units seemed to be important parameters in efficiency.

Colonization assays performed on paints demonstrated that: (i) the P(CL-VL) paints displayed a better activity against microfouling than the commercial paint; (ii) the visual inspections showed significant activity variations between paints; and (iii) one paint showed a better antimacrofouling activity than commercial paint after one year of immersion.

## Figures and Tables

**Table 1:** Composition of paints (wt %).

Composition	Paint without biocide	P(CL-VL) paints
Polyacrylic	21	-
P(CL-VL)	-	21
Xylene	32.4	32.4
Zinc pyritione	-	6
Copper thiocyanate	-	23
Additives ^1^	46.6	17.6

**^1^** plasticizer, fillers, rheological agents.

**Table 2:** Type of marine macrofouling organisms settled on substrates immersed in seawater. Ranking of the intensity and severity factors.

Surface covered by fouling (%)	Rank for intensity factor (*I*)	Type of fouling	Rank for severity factor (*G*)
No fouling	0	Biofilm	1
Up to 10%	1	Algae	3
From 10% to 20%	2	Non-encrousting species	4
From 20% to 40%	3	Encrouting species	6
From 40% to 60%	4		
From 60% to 100%	5		

The data of antifouling performance were ranked from the best to the poorest, and the significance test was conducted using one-way ANOVA method.

**Table 3:** Copolymerization of ε-caprolactone with δ-valerolactone using Sn(Oct)_2_ in bulk at 140 °C for 6 h.

Polymer	(CL/VL) ^1^	*M*_n_^2^ (g·mol^−1^)	PDI	Yield (%)	∆*H*_m_^3^ (J·g^−1^)	*T*_m_^3^ (°C)
22/78L	22/78	10,300	1.7	95	44	30
69/31L	69/31	13,200	1.6	62	36	31
82/18L	82/18	12,000	1.7	95	60	33
82/18M	82/18	25,000	1.7	90	71	36
82/18H	82/18	40,000	1.5	89	81	38

**^1^** Molar composition determined by ^1^H NMR; ^2^ Molecular weight determined by GPC, direct calibration with polystyrene standards (THF solvent); ^3^ Determined by DSC (standard deviation: 1 °C).

**Table 4:** Hydration, degradation and erosion of copolymers in paints during immersion.

Copolymer	Hydration (%) ^1^		Degradation (%) ^2^	Erosion ^3^	Zinc pyrithione release (μg/cm^2^/day) ^4^	
	14 days	8 months	8 months	8 months	to 10 days	from 10 to 45 days
22/78L	7.8 ± 0.5	9.7 ± 0.6	26	+	8	4
69/31L	20.6 ± 0.4	21.1 ± 1.0	12	++	6	4
82/18L	9 ± 0.5	16.4 ± 0.5	<1	++	6	3
82/18M	4.9 ± 0.3	12.9 ± 1.0	<1	+	6	2
82/18H	4.6 ± 0.2	14.2 ± 0.6	<1	−	7	2

**^1^** Determined by Karl Fisher titration; ^2^ Determined by GPC analysis; ^3^ Determined by touching method (−: not erodible enough, +: correct erosion: ++: too erodible); ^4^ Determined by AAS.

**Table 5:** Marine biofilm on paints after immersion in seawater.

		Paint ref.	22/78L	69/31L	82/18L	82/18M	82/18H	Negative paint
Bacteria Biomass (μm^3^/μm^2^)	6 weeks	0.160 ± 0.030	0.040 ± 0.020	0.004± 0.004	0.080 ± 0.080	0.110 ± 0.015	0.002 ± 0.002	0.008 ± 0.008
	3 months	2.070 ± 0.230	0.330 ± 0.110	0.230 ± 0.020	0.790 ± 0.190	0.310 ± 0.015	0.005 ± 0.004	n.d.
Bacteria average Thickness (μm^3^/μm^2^)	6 weeks	0.270 ± 0.090	0.060 ± 0.040	0.0002 ± 0.002	0.150 ± 0.180	0.210 ± 0.033	0.001 ± 0.001	0.010 ± 0.010
	3 months	3.700 ± 0.360	0.410 ± 0.140	0.030 ± 0.040	1.790 ± 1.210	0.050 ± 0.020	0.003 ± 0.002	n.d.
Diatom Biomass (μm^3^/μm^2^)	6 weeks	2.200 ± 0.200	7.890 ± 2.920	0.092 ± 0.080	0.240 ± 0.090	1.500 ± 0.200	0.540 ± 0.260	35.400 ± 1.600
	3 months	6.420 ± 2.350	6.480 ± 0.790	1.190 ± 0.270	0.820 ± 0.200	1.060 ± 0.150	0.340 ± 0.400	n.d.
Diatom average Thickness (μm^3^/μm^2^)	6 weeks	4.200 ± 1.200	11.830 ± 4.680	0.130 ± 0.130	0.440 ± 0.250	2.800 ± 0.500	0.330 ± 0.180	86.700 ± 13.500
	3 months	7.240 ± 2.700	9.460 ± 0.440	2.000 ± 0.010	2.300 ± 1.260	1.820 ± 0.850	0.160 ± 0.010	n.d.
Maximum Thickness (μm)	6 weeks	14.300 ± 3.900	57.490 ± 10.800	9.490 ± 6.540	28.500 ± 1.010	34.100± 4.600	15.630 ± 3.900	163.4 ± 1.000
	3 months	20.350 ± 0.500	13.740 ± 0.730	14.170 ± 3.450	12.700 ± 0.500	19.330 ± 7.190	11.330 ± 1.620	n.d.

n.d. not determined.
